# A View on the Development and Current Situation of Behavior Analysis in Europe

**DOI:** 10.1007/s42822-021-00068-w

**Published:** 2021-08-26

**Authors:** Erik Arntzen, Ricardo Pellón

**Affiliations:** 1 Department of Behavior Science, Oslo University, Oslo, Norway; 2 Departamento de Psicología Básica I, Facultad de Psicología, Universidad Nacional de Educación a Distancia (UNED), Madrid, Spain

**Keywords:** European Association for Behaviour Analysis (EABA), *European Journal of Behavior Analysis* (EJOBA), Historical perspective, Conferences, Teaching programs

## Abstract

The present article discusses essential historical trends in behavior analysis in Europe, in terms of both organizations and conferences. Of particular interest is the series of the European Meetings on the Experimental Analysis of Behaviour held in different European cities between 1983 and 2000, just when the European Association for Behaviour Analysis (EABA) and the *European Journal of Behavior Analysis* (EJOBA) both started. This article not only extends the information on EABA and EJOBA from a previous publication (Arntzen et al., 2009) but also discusses other European behavior-analytic outlets and different ways in which behavior analysis is taught in Europe.

Behavior analysis started with enthusiasm in Europe (particularly in native English-speaking countries, in keeping with its strong original inception in the United States) during the second half of the 1960s and into the 1970s. Essential for the development of behavior analysis in Europe was B. F. Skinner’s many visits—for example, to the United Kingdom, Sweden, and France. It is worth mentioning that Skinner presented at the 13th International Conference of Psychology, in 1951 in Stockholm. Several well-known psychologists, such as Jean Piaget, attended the conference. Skinner’s presentation was on “The Experimental Analysis of Behavior,” where he talked about fixed-interval and fixed-ratio schedules and how the different lengths of discriminative stimuli influenced behavior on these schedules.^1^

However, we will argue that behavior analysis did not continue to expand in the way that we might have wanted in all its domains (see, e.g., Cooper et al., 2020, and Moore, 2008, for more details about the domains). For example, we will argue that the situation for the domain of the experimental analysis of behavior is not very different from that of other countries worldwide. This article is an extension of the information about the status of behavior analysis in Europe from a previous report by Arntzen et al. (2009). The present article is focused on some organizations, conferences, and journals that have had an impact on the foundation of the European Association for Behaviour Analysis (EABA) and also the *European Journal of Behavior Analysis* (EJOBA). We will emphasize a view on the situation of behavior analysis in Europe and the role that an organization should have in fostering and sustaining collaborations between behavior analysts from the varied European regions.

## The Early European Organizations and Meetings on Behavior Analysis

As previously mentioned, the history of behavior analysis in Europe is long in terms of its world context. The British tradition of experimentation in psychology, more than in other European countries, was a good field for a promising start of behavior analysis. The Experimental Analysis of Behaviour Group (EABG), an informal organization with no membership record, was founded in 1963 in the United Kingdom, possibly instigated by Harry Hurwitz, then at Birkbeck College London before moving to the United States, and was perhaps the first organized group of people who aspired to put together and expand behavior analysis in Britain and, by extension, to the rest of Europe (see Hughes, 2007).

EABG started having 1-day meetings with a single-track program attended by less than 50 people, mainly postgraduates working in operant conditioning with pigeons or rats at different laboratories across Britain. This expanded to a pattern of two meetings each year, with a London meeting in the winter and a 2-day meeting elsewhere in the spring. In the 1980s and 1990s, respected experimental psychologists who were more aligned with associative learning joined their efforts with the EABG participants to produce meetings that were larger in attendance. The EABG was coordinated for a relatively long time by Derek E. Blackman (then at what was University College Cardiff in South Wales), and then followed successively by Christopher M. Bradshaw (then at Manchester University), Julian C. Leslie (from Ulster University), and the late C. Fergus Lowe (from Bangor University in North Wales). Since then, the EABG has been run from Bangor University, with J. Carl Hughes taking the leading role, and the conferences are now held before Easter every 2nd year (opposite years of the EABA conferences). These last decades have seen a shift in the EABG from focusing largely on operant conditioning in nonhuman animals to focusing on studies of human operant behavior and applied behavior analysis, with an emphasis on requirements for professional training. The EABG’s audiences have become, therefore, much larger and more varied—nearly 200 attended the most recent meetings, with conferences now lasting 3 days and run at University College London. The participants are drawn from a wide range of countries within and beyond Europe, which was also happening in the early days of the EABG.

From its early inception in Europe through the United Kingdom, behavior analysis expanded to different countries in the 1970s; particularly interested were the Behaviour Analysis in Ireland (BAI) group (comprising the whole island of Ireland, both the Republic of Ireland and Northern Ireland), which later became the Division of Behaviour Analysis of the Psychological Society of Ireland (see Leslie & Tierney, 2013), and the Norwegian Association for Behavior Analysis (NAFO), which was founded in 1973. The UK Society for Behaviour Analysis was established in 2013. Other examples of the development of behavior analysis in Europe can be seen in the cases of Italy (Moderato & Presti, 2006), Spain (Ruíz et al., 2006), the Czech Republic (Kingsdorf & Pančocha, 2020), and France (Richelle et al., 2006). For example, Richelle et al. (2006) described how the French-speaking parts of Europe were not as influenced by behavior analysis as the English-speaking parts had been. However, they argue that it was not because of language per se but rather because France was already “invaded” by cognitivism—and of course psychoanalysis. Truly, even in the domain of British experimental psychology, behavior analysis and behaviorism have suffered regularly from misinterpretations (Pellón et al., 2019), and the challenges surrounding the misinterpretations of behavior analysis have also been reported elsewhere (e.g., Arntzen et al., 2010; Todd & Morris, 1983). In Spain, the influence of behavior analysis was relatively well accepted initially, but then its development was subsumed under cognitive psychology (as in many other places), and certainly in Spain, the influence of psychoanalysis was not as strong as it was (and still is) in France (Ruíz et al., 2006). For a more detailed treatment of the development of behavior analysis in different geographical areas of Europe, see Pellón et al. (2019).

An important aspect of the development of behavior analysis in Europe has been the organization of international conferences, initiated by the leaders of the EABG and BAI. Perhaps the first impactful example was the symposium held in 1980 at the University of Manchester on “Recent Developments in the Quantification of Steady-State Operant Behaviour” (see Bradshaw et al., 1981), with the predominant participation of many well-established and respected behavior analysts from the United States. This meeting was followed by the first two conferences of the European Meetings on the Experimental Analysis of Behaviour (EMEAB), organized at the University of Liege in Belgium, led by Marc Richelle, and supported by an international committee. The first meeting was held in 1983 and was an extraordinary success in presenting Europe as a forceful platform for getting together for a first-time psychologist of different nationalities with varied interests in behavior analysis (see Lowe et al., 1985). Being intentionally focused, the program centered on contributions from Europe, and while successful, the prime contributor at the meeting was B. F. Skinner, accompanied by a good array of influential American behavior analysts. The title of B. F. Skinner’s presentation was “The Evolution of Behavior,” where he analyzed the roles that imitation and modeling might have had in the evolution of behavior, including our social cultural practices (see Skinner, 1984). A second EMEAB followed 5 years later, in 1988, organized similarly to the first one (Blackman & Lejeune, 1990), but was possibly less impactful in terms of attendance and overall recognition.

The third EMEAB was held in Dublin, Ireland, in 1997 and was successful, with participants coming from countries within and beyond Europe. The principal contributions at this conference (see Leslie & Blackman, 2000) represented a perhaps more conventional range of topics within behavior analysis than that of the previous EMEAB, and with substantially more contributions from English-speaking behavior analysts than at the previous meetings. However, the aftereffect was to establish a European dimension for behavior analysis, something that was taken further a few years later in a fourth, and final, EMEAB held in Amiens, France, in 2000. This last meeting took place when efforts were taken to establish a formal EABA, which from then on took the lead in organizing conferences and getting people interested in behavior analysis in Europe together. In addition, the EJOBA first appeared in 2000.

The topics covered by the different EMEABs included selectionism, behavioral and ethological approaches, the behavioral analysis of drug effects, human operant behavior, relational learning, behavior analysis and language, and behavioral medicine. The contents of the first three EMEABs were reviewed and put in a current perspective by Leslie (2013).

It should also be noted that in parallel with the emergence of the EABA, the US-based Association for Behavior Analysis International (ABAI; then simply ABA) held its first conference within Europe in Venice, Italy, in 2001, which was preceded by one of the series of International Congresses on Behaviorism and the Sciences of Behavior, organized in Seville, Spain, in 1998, which greatly informed discussions regarding the constitution of the EABA. Thus, by the early 2000s, behavior analysis appeared to have established a secure presence within a broad and diverse international framework beyond the United States and countries such as Mexico and Brazil, particularly in Europe. The series of international ABAI conferences has recurrently been held in Europe: Oslo (Norway) in 2009, Granada (Spain) in 2011, Paris (France) in 2017, and Stockholm (Sweden) in 2019; they will continue in 2022 with a conference in Dublin (Ireland), which was postponed from 2021 due to the coronavirus pandemic affecting the world at this moment. Similarly, the series of International Congresses on Behaviorism and the Sciences of Behavior (assembled by Peter Harzem together with Emilio Ribes) were also frequently held in Europe: Palermo (Italy) in 1994, Seville (Spain) in 1998 (as mentioned before), and Santiago de Compostela (Spain) in 2006. These were continued under the name International Congresses on Behavior Studies (and under the leadership of Paolo Moderato) in Rethymno (Crete, Greece) in 2010, Lisbon (Portugal) in 2012, and Milan (Italy) in 2014.

Another series of conferences that intertwined with the aforementioned and contributed to spreading behavior analysis in Latin European countries was the international congresses on behavioral studies called Latini Dies that connected Spain, France, Mexico, Portugal, and Italy. Those who met at these conferences influenced the creation of, in 1992, the common scientific journal *Acta Comportamentalia*, published in Mexico, which hosts articles in Spanish, French, Italian, and Portuguese.

Finally, verbal relations were the main topic at three international meetings. The first was held in Kreuznach, Germany, in 1986, and the others in Mexico in 1988 and in Brazil in 1989. The presentations for the first meeting in Germany were edited by Hayes and Chase (1990).

## The EABA and Its Conferences

The EABA was officially founded at the 2001 ABAI international conference in Venice, after a number of discussions that took place at meetings in London (EABGs 1999, 2000, and 2001), Dublin and Amiens (third and fourth EMEABs, in 1997 and 2000), and particularly Seville in 1998 at the first focused meeting during the Fourth International Congress on Behaviourism and the Sciences of Behaviour. Arntzen et al. (2009) gave detailed information about those discussions at the turn of this century, and the timeline at https://www.europeanaba.org/index.php/about-us/eaba-history shows important events in the history of the EABA. Further information on the EABA can be found on its official website.^2^

Beyond the EABA, there are several national organizations in Europe (see Table 1). We have also listed a number of larger organizations in different countries. The list is based on information from the ABAI and direct contact with leading behavior analysts in different countries.

**Table 1:** Overview of different organizations and groups, national or local, and websites for behavior analysis in Europe

Organization	Website
ABA España	https://aba-elearning.com
ABA Germany	https://www.aba-d.de/
ABA of Italy	http://www.iescum.org/doceboCms/
ABA Switzerland	https://www.abaswitzerland.ch
ABA Italy	https://www.abaitalia.org/
Association for the Advancement of Radical Behavior Analysis	https://www.aarba.eu
Association Française—Les Professionnels de l’Analyse du Comportement	https://www.onpac.fr
Baltic Association for Behavior Analysis	http://www.findglocal.com/LV/Riga/130342304475704/Baltic-Association-for-Behavior-Analysis
Behaviour Analysis in Ireland	https://www.psychologicalsociety.ie/groups/Division-of-Behaviour-Analysis
Czech Association of Applied Behavior Analysis	https://csaba.cz/en/
Experimental Analysis of Behaviour Group UK and Europe	http://eabg.bangor.ac.uk/
Finnish ABA	https://versokuntoutus.fi/verso-kuntoutus/aba-kokonaiskuntoutus-applied-behavior-analysis/
French ABA	
Icelandic ABA	https://aferli.is/
Hellenic ABA	https://www.behaviorism.panteion.gr/index.php/en/
Norwegian ABA	https://www.atferd.no/
Polish Association of Behavioral Therapy	https://eabct.eu/about-eabct/member-associations/poland-pacbt/
Polish Society for Behavioral Psychology	http://behawioryzm.pl
Society for the Advancement of the Scientific Study of Behavior	https://savecc.com/
Swedish ABA	https://www.swaba.se/

Arntzen et al. (2009) described the development of the first four EABA conferences: Parma (Italy) in 2003, Gdansk (Poland) in 2005, Milan (Italy) in 2006, and Madrid (Spain) in 2008. As a way of quantitative estimation, they compared the number of oral presentations between the meetings in Gdansk and Madrid, which showed an overall increase from 57 to 105, with most topics covered at both conferences. The present article outlines details from the conferences that followed: Crete (Greece) in 2010, Lisbon (Portugal) in 2012, Stockholm (Sweden) in 2014, Enna (Italy) in 2016, and Würzburg (Germany) in 2018. The 10th conference, in Tampere (Finland), scheduled for 2020, was postponed to 2022 due to the coronavirus pandemic; it will be held in Tampere from June 15 to 18, 2022. Details are available at https://events.tuni.fi/eaba2022/.

The EABA arranges a conference every other year. The venue is decided by applications to host the conference. Holding such a conference is part of the main goal of the EABA, which is to encourage and spread behavior analysis in Europe. Thus, the content at the different conferences focuses on the domains of behavior analysis, including theoretical and conceptual, experimental, and applied contributions within behavior analysis (e.g., Cooper et al., 2020). In addition to the scientific program, an assembly meeting is scheduled during the conferences, and all local organizers have included social events. The conferences have been attended by people interested in behavior analysis representing different countries all over Europe and many places outside Europe.

### Crete (2010)

This conference was at the University of Crete, Greece. The scientific program included 37 events: 20 paper sessions, 11 symposia, and 6 keynote addresses. In addition, 25 posters were exhibited, and a presidential address served as the opening of the conference. Invited presenters were Gina Green (*Implementing Evidence-Based Guidelines for Autism Treatment*), Giulio Lancioni (*Assistive Technology for Behavioural Interventions for Persons With Severe, Profound and Multiple Disabilities*), Julian Leslie (*Animal Models of Psychiatric Disorders: Behaviour Analysis Perspectives*), Maria Malott (*ABAI and International Developments*), Jerry Shook (*The Behavior Analyst Certification Board and International Credentialing*), and Richard Shull (*Bouts, Changeovers and Operant Units*).

### Lisbon (2012)

The conference venue was at both the University of Lusíada and the Cultural Centre of Belem, Portugal. Altogether, the scientific program included 37 events: 12 symposia, 18 paper sessions, and 7 keynote addresses. In addition, a regular poster session with 65 posters and 8 Expo posters was held, and a presidential address served as the opening of the conference. Invited speakers were Jennifer Austin (*School-Based Functional Analyses With Typically Developing Children: Considerations for Research and Practice*), Chris Bradshaw (*Some Recent Work on the Behavioural and Neural Basis of Inter-temporal Choice*), Bill Heward (*Climate Change and the Global Need for Sustainable Practices: Opportunity, Challenge, and Responsibility for Behaviour Analysis*), Peter Killeen (*The Five Causes of ADHD*), Robert Mellon (*Transitions to Safety in Avoidance: Experimental Findings and Clinical Implications*), Paolo Moderato (*Behaviour Analysis of Human Behaviour: The 4 P’s Analysis*), and Gabríela Sigurðardóttir (*Some Thoughts and Concerns About the Education of Behaviour Analysts*).

### Stockholm (2014)

The conference venue was at Stockholm University, Sweden. The scientific program included 35 events: 16 paper sessions, 14 symposia, and 6 keynote addresses. In addition, a poster session with 49 posters and 14 Expo posters was held. Invited speakers were Shahla Alai-Rosales (*Advising an Experimental Thesis in Applied Behavior Analysis: A Data-Based Program Description*), Mecca Chiesa (*Implications of the Conceptual Analysis of Behaviour for the Future of Behaviour Analysis*), Camille Ferond (*Organizational Influence*), Iver H. Iversen (*The Importance of Basic Research for Successful Application of Behavior Analysis*), John C. Moore (*Why Study Radical Behaviorism as Philosophy*?), and Niklas Törneke (*Relational Frame Theory for Clinical Use*). A presidential address served as the opening of the conference.

### Enna (2016)

The conference was hosted at Kore University of Enna, Sicily, Italy. Altogether, the scientific program included 55 events: 25 symposia, 24 paper sessions, 1 panel discussion, and 5 keynote addresses. In addition, a poster session, opening session, and presidential address were a part of the program. The invited speakers were Angelika Anderson (*Technology-Assisted Interventions to Teach Work Skills to Adults With Autism Spectrum Disorders*), Lanny Fields (*How Meaningful Stimuli Enhance Equivalence Class Formation*), Wayne Fuqua (*Improving the Effectiveness and Accountability of ABA Service Delivery Through Evidence-Based Practice Strategies*), Ricardo Pellón (*How Reinforcement Theory Can Inform Us About Problematic Behavioral Excess*), and Alan Poling (*Extending the Scope of Applied Behavior Analysis: APOPO as a Case Study*).

### Würzburg (2018)

The conference was co-organized by the Faculty of Applied Social Sciences and School of Social Work at the University of Applied Sciences, Würzburg, Germany. The conference’s scientific program included 48 events: 22 paper sessions, 18 symposia, 2 panel discussions, and 6 keynote addresses (a couple of the keynotes had to be canceled on short notice for different reasons). In addition, the conference included one poster session with 26 regular posters and one Expo session with 5 posters, as well as an opening session and a presidential address. The invited speakers were Sigurður Óli Sigurðsson (*Behavioral Safety: Research and Practical Considerations*), Nancy Marchand-Martella (*When It Comes to Best Practices in Instruction, Remember to Be Like Kevin Bacon!* ), Carol Pilgrim (*Translational Research and Stimulus Equivalence*), James Todd (*How We Got Here and Why We Probably Should Have Looked Out the Window on the Way: Some Personal Reflections on the Current Status of Behavior Analysis—With Pictures*), Maurice Feldman (*Behavior Analysis in Child Welfare: Contextual Behavioral Assessment and Intervention to Prevent Child-Neglect in At-Risk Families*), and Mark Mattaini (*Behavioral Systems Science for Social Action*).

## Behavior-Analytic Outlets

In this section, we will present different journals on behavior analysis published in Europe. We will also include some details about the number of publications per year and the types of special issues published in EJOBA.

### EJOBA

EJOBA was founded in 2000, and the troika composed of Erik Arntzen, Per Holth, and Arne Brekstad served as editors. Since 2014, Erik Arntzen has served as the editor-in-chief. At the same time, several scholars have served as associative editors. The journal has published two issues each year for more than 20 years. The NAFO has economically supported EJOBA in this period. Starting in 2015, EJOBA has been published by Taylor & Francis Group in collaboration with the NAFO. Nevertheless, the NAFO is still the owner of EJOBA.

The primary purpose of EJOBA is to publish experimental reports and theoretical/conceptual articles within behavior analysis. In addition, review articles will also be considered for publication. Some issues will be special issues, with articles, for example, based on presentations at different conferences. All submitted manuscripts are subject to initial appraisal by the editor and, if found suitable for further consideration, to peer review by independent, anonymous expert referees. All peer review is double-blind, and submission is made via Editorial Manager. Papers can be submitted via the Editorial Manager website at https://www.tandfonline.com/loi/rejo20.

**Fig. 1:**
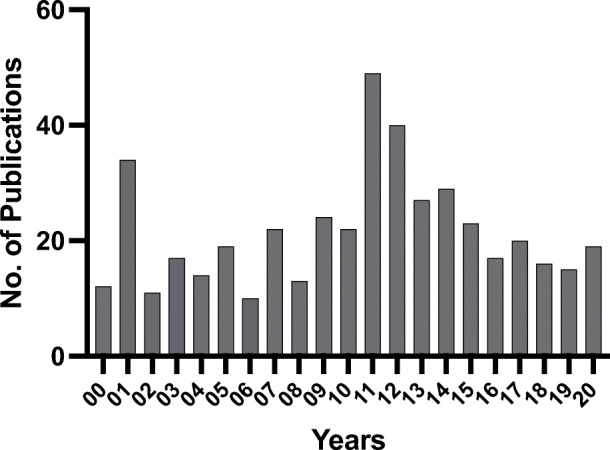
Published articles in the European journal of behavior analysis

EJOBA has focused on publishing issues with a target article followed by commentaries, as well as articles based on presentations from the two main conferences in behavior analysis in Europe (those organized by EABG and EABA; see Table 2). Table 2 gives an overview of the main content in the two issues each year. Special issues have been published four times for the EABA conferences, with a total of 53 articles, and four times for the EABG conferences, with a total of 33 articles. A total of 453 papers have been published from 2000 to 2020 (see Fig. 1). The mean is 21.6 articles published per year with a peak of 49 in 2011. The large number of articles in 2011 was due to publishing two special issues and the reprinting of the Sidman “remarks.”

**Table 2:** An overview of issues including special issues and conferences

Year	Issue	Special issue/special section	Conference or meetings
2000	1–2	Behaviourism	
2001	1	Stimulus equivalence	
2001	2	Bereavement and grief	
2003	1–2	Precision teaching	
2004	2	Skinner tribute	
2005	1	Noncontingent reinforcement	
2009	2		EABG 2009
2010	2		Summit 1 in Texas
2011	1		EABA 2011 in Crete and Sidman “remarks”
2011	2		EABG 2011
2012	1		Sarasota 2011 (1st)
2012	2	Flight from the experimental analysis of behavior	
2013	1		Sarasota 2013 (2nd)
2013	2		EABA 2012 in Lisbon and EABG 2013
2014	1	Ulman–Skinner letters	
2015	2		EABA 2014 in Stockholm
2016	1		Sarasota 2016 (3rd)
2016	2		EABG 2015
2017	2		Sarasota 2017 (4th)
2019	2		Sarasota 2018 (6th)
2020	1		Summit 2 in Stockholm
2020	2		EABA 2018 in Würzburg

*EABG* Experimental Analysis of Behaviour Group, *EABA* European Association for Behaviour Analysis

Over the years, EJOBA has published several target articles, including commentaries. For example, in the special issue on stimulus equivalence (Issue 1 in 2001), the target article was written by Tonneau (2001a). The content of the article created considerable discussion: 19 commentaries and the reply (Tonneau, 2001b). Also, later articles continued the discussion raised in this special issue. Another example is the reprinted article on “Flight From Experimental Analysis” by Catania (2012), which evoked thought-provoking commentaries.

Organizers of symposia or meetings other than EABG and EABA have contacted EJOBA to ask for possible publication of special issues or sections. Special sections have been published based on meetings in Sarasota, Florida, and from two International Summits on Higher Education, Autism, and Behavior Analysis, the first in Texas in 2010 (Ala’i-Rosales et al., 2010) and the second in Stockholm in 2019 (Roll-Pettersson, Dillenburger, et al., 2020a). The symposia in Sarasota were organized by Iver Iversen and Per Holth to honor Murray Sidman. Sidman lived in Sarasota and attended the meetings. The idea for these meetings was to present papers on methodological and conceptual issues. For example, from the first meeting, a paper on sculpture and behavior analysis was published (Constantine, 2012), and from the second meeting, a paper on measuring behavioral changes in Alzheimer’s patients was published (Sidman, 2013). A total of 26 papers were published from five of the six meetings in Sarasota. From the two summits, 18 papers were published.

#### From Behaviour Analysis Letters to Behavioural Processes

**Fig. 2:**
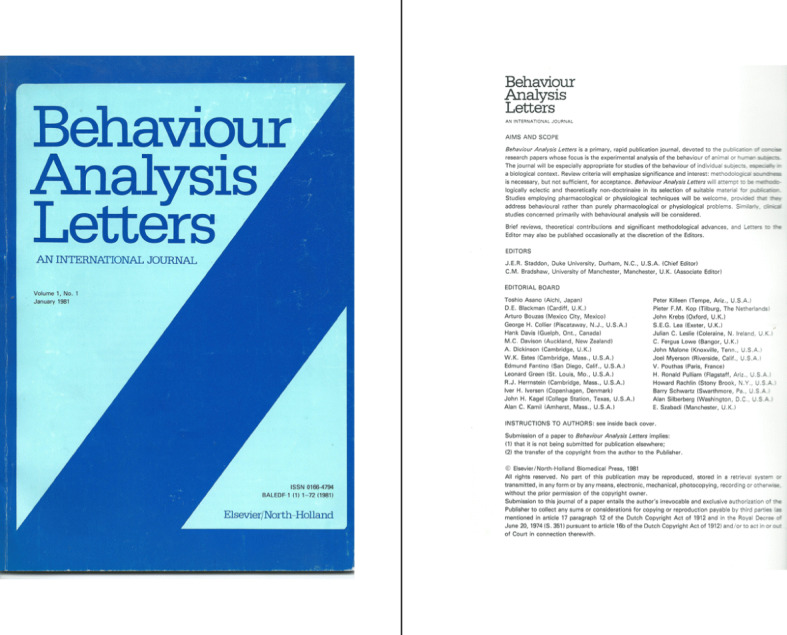
The cover of the first issue of Behaviour Analysis Letters, along with the appointed editors and the first editorial board. *Note.* By courtesy of Julian C. Leslie, who served as a member of the editorial board in 1981

The increasing momentum of behavior analysis in Europe during the 1980s (see the previous discussion) can be seen as well in terms of the foundation of the first behavior-analytic journal within a European domain. This was *Behaviour Analysis Letters*, published by Elsevier/North-Holland Biomedical Press. It only lasted 3 years, from 1981 through 1983. Contributors to the first issue were mostly behavior analysts from native English-speaking countries (with the exception of Iver Iversen, then at the University of Copenhagen, Denmark), with Fergus Lowe (Bangor University) and John Wearden (then at Manchester University) as contributors from the United Kingdom. Figure 2 shows the cover of the first issue of *Behaviour Analysis Letters* along with the appointed editors and the first editorial board; they were behavior analysts mainly from the United Kingdom and the United States, as was the case for each one of the two editors.

*Behaviour Analysis Letters* was subsumed in 1984 into *Behavioural Processes*, another Elsevier journal that was first published in July 1976. *Behavioural Processes* nowadays, as stated on its webpage (https://www.journals.elsevier.com/behavioural-processes), is dedicated to the publication of research on animal behavior from any theoretical perspective—including the behavior-analytic, cognitive, ethological, ecological, and evolutionary points of view—and considers publications investigating basic behavioral phenomena and behavioral studies of more applied significance. The inclusion of the behavior-analytic perspective was due to the incorporation of the policies that came from *Behaviour Analysis Letters*, and indeed John E. R. Staddon (Duke University, coming from *Behaviour Analysis Letters*) was appointed editor of the journal in 1984, together with Georges Thinés (Catholic University of Louvain), who had been acting as the sole editor since the inception of the journal in 1976. *Behavioural Processes* still preserves that double editorship, with corresponding associate editors from the two broad approaches of cognitive neuroscience and behavior analysis.

#### Behavioural Pharmacology

As with the attempt to establish a behavior-analytic journal in the early 1980s, there was also a fruitful attempt to establish a journal for the growing field of behavioral pharmacology, on which behavior analysis was of a key influence. Indeed, in fall 1989, the first issue of *Behavioural Pharmacology* was published; the first editors were Paul Wilner (Swansea University, United Kingdom), Michael Emmet-Oglesby (Tufts University, United States), and David Sanger (Sanofi-Synthélabo, France), for years allied with the European Behavioural Pharmacology Society, established a few years earlier, in 1986:

The first editorial of *Behavioural Pharmacology* was clear in its aims:

While there is no shortage of publication outlets for high quality research in behavioural pharmacology, we believe that there is a need for a journal that recognizes and addresses the complexity of behaviour as its primary focus. . . . Our object in launching *Behavioural Pharmacology* is to provide a forum in which behaviour and pharmacology are equally prominent. (Willner et al., 1989, p. 1)

The current editor is Louk Vanderschuren (Utrecht University, Netherlands), with Paul Willner still involved as one of its associate editors. *Behavioural Pharmacology* publishes reports in areas “ranging from ethopharmacology to the pharmacology of schedule-controlled operant behaviour, provided that their primary focus is behavioural” (Willner et al., 1989, p. 1).

### Some Other Behavior-Analytic Outlets

In Europe, other behavior-analytic journals have been successful. The *International Journal of Psychology and Psychological Therapy / Revista Internacional de Psicología y Terapia Psicológica*, published in Spain and accepting contributions both in Spanish and in English, was established in 2001. It is mainly run from the University of Almería and is an interdisciplinary journal that publishes empirical and theoretical contributions in any area of psychology, with a stronger emphasis on applied behavior analysis and applied psychology. *Behavioral Psychology / Psicología Conductual* is a journal established in 1993 that is operated from the University of Granada, covering behavioral studies broadly understood, with a particular emphasis on clinical psychology. Finally, *Conductual* is a free-access electronic journal established in 2013 and run by a mixture of Spanish and American (predominantly Mexican) behavior analysts; it was initially more in the interbehaviorism orbit, but that slowly shifted to a more conventional behavior-analytic tradition, linked in different ways to the Sociedad para el Avance del Estudio Científico del Comportamiento since 2018, a Spanish behavioristic society, as well as to the Seminario Internacional sobre Comportamiento y Aplicaciones, an organization based in Mexico. *Conductual* publishes conceptual, basic, and applied articles in Spanish, Portuguese, and English, all with an emphasis on the study of the behavior of individuals based on empirical evidence or shared philosophical postulates to natural sciences. Similarly, as mentioned previously, *Acta Comportamentalia* holds close objectives.

## Teaching Programs for Behavior Analysis

**Fig. 3:**
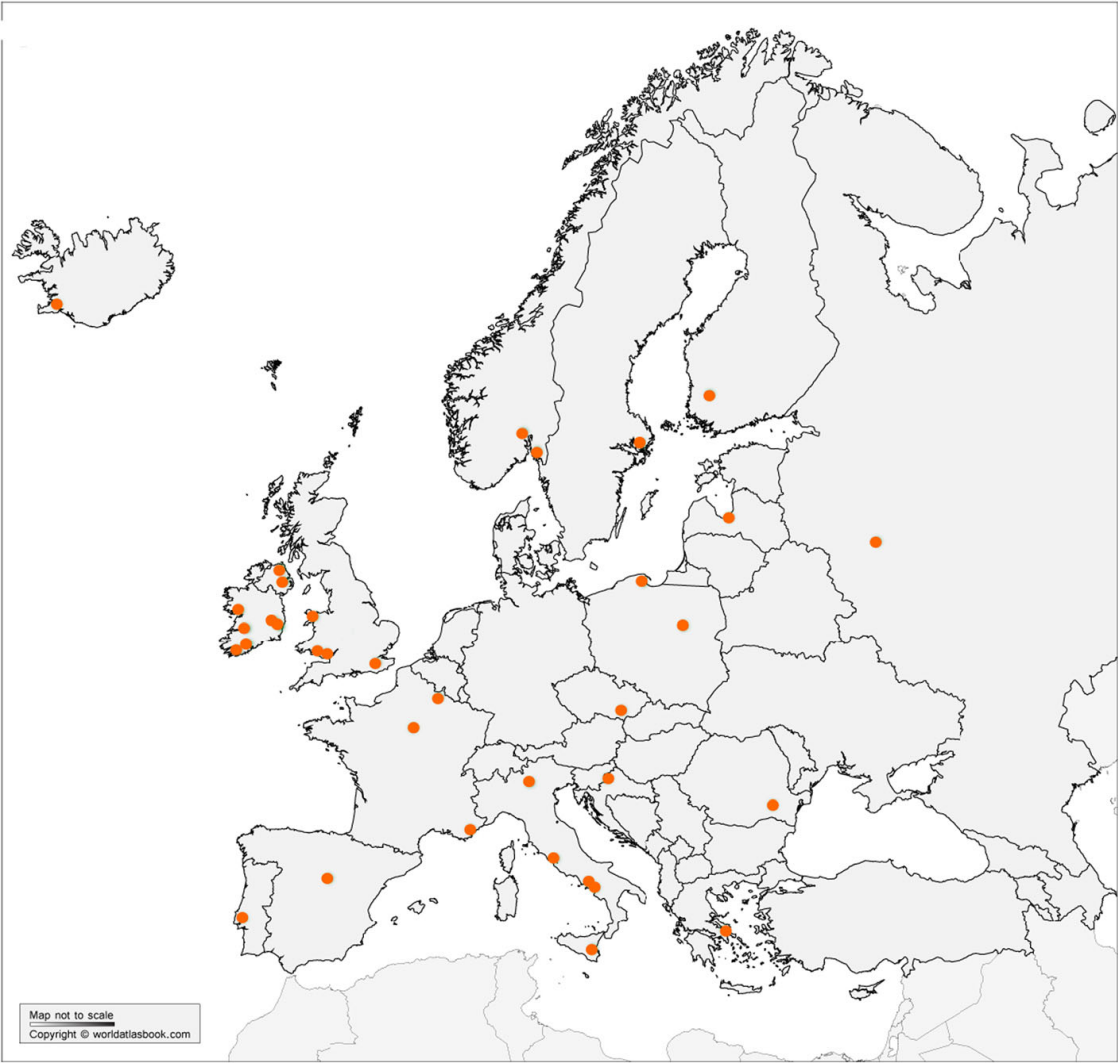
An overview of behavior analysis training programs in Europe

Figure 3 shows the locations of different universities and university colleges that have programs for teaching behavior analysis (based information presented in Steingrimsdottir et al. (2019)). The programs are spread over most of Europe. It can be difficult to define what such a program should include, but in this case, we have not counted instances in which there are only some workshops with a focus on behavior analysis.

It is also essential to mention the types of teaching of behavior analysts that occur over more intensive periods—for example, summer schools in Oslo after the ABAI conference in 2009 and three summer schools arranged by the EABA in Rethymno (Crete, Greece) in 2015, Cádiz (Spain) in 2017, and Pelči (Latvia) in 2019. In general, the scientific program at these different summer schools has included scholars from different parts of Europe and the United States and students (the vast majority, if not only, European) interested in behavior analysis.

Sigurdardottir (2013) discussed some historical changes in teaching behavior analysts and also some issues from when the Behavior Analyst Certification Board (BACB) was established. Additionally, several papers have discussed the role of the BACB in Europe (e.g., Arntzen et al., 2009; Hughes & Shook, 2007; Virués-Ortega et al., 2009). Recently, Martin and Carr (2020) presented an update on the certification of behavior analysts in Europe. They found 34 institutions with verified course sequences (VCSs) across Europe, in 19 countries. Furthermore, the United Kingdom has 5 VCSs, and 10 countries have one institution with a VCS. As Martin and Carr discussed in that article, the system of VCSs is not directly related to the quality of the teaching but rather is a way to ensure a common process of requirements for applying for the BACB’s certification. The system of BACB certification has been important for the development of training programs worldwide. The United Kingdom has the greatest number of BCBA certificants in Europe, with 552 people.

However, from January 1, 2023, only residents of the United States, Canada, and Australia will be able to apply for BACB certification (residents of the United Kingdom may apply through 2025). The BACB provided an argument for the change: “The rationale behind changing our international focus is multifaceted and was shaped over a multiyear period following extensive research, training from the certification industry, and consultation with certification and testing experts” (https://www.bacb.com/wp-content/uploads/2020/06/Recent-Changes-to-International-Focus_200520.pdf). As noted by Roll-Pettersson, Gena, et al. (2020b), the information about this change came as a big surprise and was totally unexpected. Hence, it seems important to establish certification systems in Europe, either in separate countries or as a collaboration between countries.

## Summary

Behavior analysis in Europe has some strong historical roots. The present article showed that behavior analysis in Europe is widely (but not yet sufficiently) spread across different countries. Furthermore, the outlets we presented in the paper are important for students, junior researchers, and senior researchers, and clinicians, so they may publish their research and interventions in a variety of peer-reviewed journals. We would argue that these peer-reviewed journals are essential methods of dissemination of behavior analysis in Europe. The two main behavior-analytic conferences in Europe, the EABG’s and EABA’s, are as important as the journals for the dissemination of behavior analysis in Europe, with the EABA conferences serious about including all the domains of behavior analysis in their scientific programs.

## Footnotes

1 Based on information from Erik Arntzen’s visits to the Pusey Library at Harvard in Cambridge, Massachusetts. The Pusey Library has archived 82 boxes of material from B. F. Skinner’s work.

2 https://www.europeanaba.org
